# Daytime sleepiness in elementary school students: the role of sleep quality and chronotype

**DOI:** 10.11606/s1518-8787.2021055004124

**Published:** 2022-06-27

**Authors:** Tâmile Stella Anacleto, João Guilherme Fiorani Borgio, Fernando Mazzilli Louzada

**Affiliations:** I Universidade Federal do Paraná Laboratório de Cronobiologia Humana Programa de Pós-Graduação em Biologia Celular e Molecular Curitiba Paraná Brasil Universidade Federal do Paraná. Laboratório de Cronobiologia Humana. Programa de Pós-Graduação em Biologia Celular e Molecular. Curitiba, Paraná, Brasil; II Universidade Federal do Paraná Departamento de Medicina Forense e Psiquiatria Curitiba Paraná Brasil Universidade Federal do Paraná. Departamento de Medicina Forense e Psiquiatria. Curitiba, Paraná, Brasil; III Universidade Federal do Paraná Laboratório de Cronobiologia Humana Departamento de Fisiologia Curitiba Paraná Brasil Universidade Federal do Paraná. Laboratório de Cronobiologia Humana. Departamento de Fisiologia. Curitiba, Paraná, Brasil

**Keywords:** Adolescent, Sleep, Circadian Rhythm, Quality of Life, Activities of Daily Living, Education

## Abstract

**OBJECTIVE:**

To investigate the occurrence of daytime sleepiness and associated sleep factors in a sample of elementary school students who attended school in the afternoon schedule.

**METHODS:**

Sleep data from 363 Brazilian public school students (12.78 ± 1.36 years, 206 girls) were obtained by applying questionnaires in classrooms. All subjects attended school in the afternoon schedule, with classes starting between 1:00 and 1:20 p.m. Daytime sleepiness was assessed by the pediatric daytime sleepiness scale; sleep quality, by the mini-sleep questionnaire; and sleep patterns and chronotypes, by the Munich chronotype questionnaire. Scores equal to or greater than 15 pediatric daytime sleepiness scale points were considered as excessive daytime sleepiness. The predictive power of sleep variables on daytime sleepiness was evaluated by a multiple linear regression.

**RESULTS:**

The subjects in the sample had an average time in bed greater than nine hours both on school days and on weekends. Nevertheless, 52.1% had an average pediatric daytime sleepiness scale score equal to or greater than 15 points, indicative of excessive daytime sleepiness. As for their quality of sleep, 41.1% had a very altered sleep. We observed, by a multiple linear regression, that quality of sleep (β = 0.417), chronotype (β = 0.174), mid-sleep on school days (β = 0.138), and time in bed (β = - 0.091) were all significant in predicting daytime sleepiness.

**CONCLUSION:**

This study showed the occurrence of excessive daytime sleepiness in non-sleep deprived students who attended school in the afternoon. The worst quality of sleep and eveningness had a greater predictive power on daytime sleepiness than time in bed. Therefore, we must consider other factors in addition to sleep duration when planning interventions for daytime sleepiness.

## INTRODUCTION

The literature has well documented the incompatibility between adolescents’ sleep patterns and morning school schedules^1,2^. Its more evident consequence is chronic sleep deprivation associated with a negative impact on academic performance^3,4^. Already discussed and implemented countermeasures have focused on reducing these undesirable consequences^5^. Basically, we can see two main trends: sleep education^6^ and changes in school start times^7^. School-based sleep education programs can improve sleep habits, but most produce only short-term benefits^8^. The need to delay school start times have become a consensus among sleep researchers^1,9,10^, a concern which has also grown in public policy discussions^11^. Alternatively, switching classes from mornings to afternoons also seems to benefit students’ sleep. Mello et al.^12^ (2001) showed a one-hour increase on sleep duration in adolescents after the transition from morning to the afternoon schedule. Anacleto et al.^13^ (2014) found similar results when comparing preadolescent children attending morning and afternoon classes. Recently, Carvalho-Mendes et al.^14^ (2020) have shown that students attending evening classes (starting at 12:30 p.m.) had greater sleep duration than those attending morning classes (starting at 7:00 a.m.).

One of the consequences of reduced hours of sleep is daytime sleepiness^15^. Pereira et al.^16^ (2015) pointed out that a short sleep duration is the main predictor of excessive daytime sleepiness (EDS) in Brazilian students. However, other studies have shown that daytime sleepiness is not only associated with sleep duration. In healthy individuals, other factors, such as poor sleep quality^17^, low stress control, and lower self-rated health^18^, as well as physical activity habits, use of social media, and consumption of processed foods^19^, seem to be associated with the perception of daytime sleepiness.

In Brazil, classes are divided into schedules, and students who attend school in the afternoon do not usually have their sleep duration affected by school hours. However, throughout pedagogical practice, we can observe the occurrence of daytime sleepiness in students who attend school in the afternoon. We designed this study encouraged by the results of previous studies and by the reports of teachers who observed the occurrence of daytime sleepiness in students who apparently have regular sleep regimens. This study aimed to investigate the occurrence of daytime sleepiness and its associated sleep factors in a sample of elementary school students who attended school in the afternoon schedule.

## METHODS

### Subjects

The sample consisted of 363 subjects (206 girls), aged 10–16 years (mean 12.78 ± 1.36 years), of which most were aged between 11 and 14 years (86.7%). All subjects were elementary school students from 15 different public schools in Curitiba, in the state of Paraná, in Southern Brazil. As for their ethnic distribution, 52.7% of students recognized themselves as white, 43.1%, as black, and 4.2% as indigenous or Asian. Most students reported belonging to families with average family incomes (B2)^20^, and 5.5% working. All subjects attended school in the afternoon schedule, with classes starting between 1:00 and 1:20 p.m., depending on the school.

### Sample Selection, Data Collection, and Analyzed Variables

The data analyzed in this study were extracted from a database collected in 2014 (March to October), which were part of a broader survey that evaluated the sleep pattern of students who attended school in the morning and afternoon. This research was designed with the aim of obtaining a wide and distributed sample of Curitiba students. For this purpose, two schools from each of the nine teaching regions of the municipality were chosen by drawing lots, which totaled 18 schools located in various central and peripheral neighborhoods. For this study, only data from subjects who attended school in the afternoon were analyzed. Out of the 18 schools visited, one had classes only in the morning and another two requested that collections must be performed only with students in the morning schedule. Thus, the subjects who made up the sample of this article came from 15 different schools in the city.

This research was approved by the ethics committee in research of the Universidade Federal do Paraná (number 504.532).

### Data Collection and Analyzed Variables

The classes in which data were collected were those indicated by the board and the pedagogical team of each school. These classes were initially visited by the researchers and all students present at that time were invited to participate in the study, after having been instructed that participation would be voluntary, that data would be obtained by individual responses to a questionnaire, and that all data collected would be kept confidential. All students who were interested in participating in the study received an informed consent form. The next day, data collection was performed and all students who were present in the classrooms and who had the consent forms signed by their parents could participate. All data (sociodemographic, and related to work, health, substance use, and sleep patterns) were obtained by self-report questionnaires filled out in the classrooms under the supervision of the experimenter. All questionnaires answered by students were collected, and the following exclusion criteria were applied: users of medication that could affect their sleep/wake cycle, users of psychoactive drugs, diagnosed psychiatric disorders, and incorrectly filled out questionnaires.

Data on work, health, and substance use were obtained by the questions:

*“Do you work? (Yes/No). If so, how many hours per day? How many days a week?”*

*“Do you have any health problems? (Yes/ No). If so, what?”*

*“Do you take any medications? (Yes/No). If so, what?”*

Three instruments were used to evaluate sleep patterns:

1. The Munich chronotype questionnaire (MCTQ) by which sleep and wake up times were obtained during school days and weekends. From these variables, time in bed and mid-sleep (the average point between the time of going to bed and waking up) were estimated. Social jetlag was estimated by the difference between mid-sleep on weekends and on school days. MSFsc (the average sleep point corrected by sleep deprivation on school days) was estimated following Roenneberg et al.^21^ (2004), and was considered as an estimate of subjects’ chronotypes.
2. The pediatric daytime sleepiness scale (PDSS), translated and validated for Brazilian Portuguese^22^, by which daytime sleepiness was assessed. This scale, composed of eight items, generates a score varying from 0–32 points, in which higher scores reflect higher daytime sleepiness. According to what was proposed by Meyer et al.^23^ (2018), scores equal to or greater than 15 PDSS points were indicative of EDS.
3. The mini-sleep questionnaire (MSQ), translated^24^ and validated^25^ for Brazilian Portuguese, by which a scale composed of 10 items generates scores that vary between 10 and 70 points. The MSQ score can categorize sleep quality into four levels: good sleep (10–24 points), slightly altered sleep (25–27 points), moderately altered sleep (28–30 points), and very altered sleep (≥ 31 points)^24^.

Except for mid-sleep on school days, the investigated variables showed a non-parametric distribution. Differences between school days and weekends on the average sleep time, wake up time, time in bed, and mid-sleep were compared by the Wilcoxon test. Correlations between PDSS scores, age, and sleep variables were estimated by the Pearson’s correlation test. To evaluate the variables that could be predictive of daytime sleepiness, a hierarchical multiple logistic regression analysis was performed. The PDSS score was used as a dependent variable, whereas the MSQ score, the MSFsc, mid-sleep on school days, and time in bed were used hierarchically as independent variables.

Data were analyzed in the IBM SPSS Statistics software, version 20.0. For all analyses, a significance probability of 5% was considered.

## RESULTS

The sample consisted of adolescents who maintained an average time in bed longer than nine hours both on school days and weekends (Table 1). We found a delay in the time of going to bed and waking up on weekends without any variation in total time in bed. The magnitude of social jetlag in the sample was 1.43 ± 1.26h.

**Table 1:** Sleep variables during school days and weekends for the total sample.

	School days	Weekends	Z; p
Sleep time^a^	0:01 (1:58)	1:47 (2:11)	14.564; < 0.001^d^
Wake up time^a^	9:18 (1:52)	10:62 (1:75)	13.199; < 0.001^d^
Time in bed^b^	9.17 (1.46)	9.16 (1.71)	0.372; 0.710
Mid-sleep^b^	4.59 (1.37)	6.00 (1.65)	14.996; < 0.001^d^
PDSS score^c^	14.65 (6.01)		
EDS (%)	52.1		
MSQ score^c^	29.30 (8.40)		
Good sleep - MSQ (%)	29.0		
Social jetlag^a^	1.43 (1.26)		

PDSS: pediatric daytime sleepiness scale; EDS: excessive daytime sleepiness; MSQ: mini-sleep questionnaire.

Note: mid-sleep: the average point between the time of going to bed and waking up; Social jetlag: estimated by the difference between the mid-sleep on weekends and on school days.

^a^ Averages (SD) are indicated in hours and minutes.

^b^ Averages (SD) are indicated in hours.

^c^ Average (SD).

^d^ Wilcoxon test , p < 0.05.

We found an average PDSS score of 14.65 (6.01) points, and 52.1% of the students in the sample showed EDS. The average MSQ score was 29.3 (8.4), and 29% of subjects had scores compatible with good sleep quality. We also observed that 41.1% of students had scores indicative of very altered sleep.

We found that 5.5% of students reported working. Among students who recognized themselves as workers, their average daily working time was 3.89 (2.08) hours and the average number of days worked during the week was 3.4 (1.93) days.

We found by a correlation analysis (Table 2) that PDSS scores correlated with MSQ scores (r = 0.471; p < 0.001), the MSFsc (r = 0.362; p < 0.001), and mid-sleep on school days (r = 0.35; p < 0.001). Thus, we can claim that the greater the daytime sleepiness, the worse the quality of sleep and the greater the tendency for eveningness. We also observed correlations among PDSS scores, time in bed (r = - 0.124; p = 0.019), and social jetlag (r = 0.148; p = 0.005). Thus, the shorter the time spent in bed and the greater the social jetlag, the greater the daytime sleepiness.

**Table 2:** Pearson’s correlation tests between PDSS score, age, and sleep variables.

Variables	r	p
Age	0.061	0.250
MSQ	0.471	< 0.001^a^
Time in bed (school days)	- 0.124	0.019^a^
Mid-sleep (school days)	0.350	< 0.00^a^
Social jetlag	0.148	0.005^a^
Chronotype (MSFsc)	0.362	< 0.001^a^

PDSS: pediatric daytime sleepiness scale; MSQ: mini-sleep questionnaire.

Note: mid-sleep on school days: the average point between the time of going to bed and waking up on school days; Social Jetlag: estimated by the difference between mid-sleep on weekends and on school days; MSFsc: was considered as an estimate of subjects’ chronotypes.

^a^ p < 0.05.

Considering the results found, we performed a hierarchical multiple linear regression analysis which included PDSS scores as its dependent variable (Table 3). We sequentially inserted MSQ scores, the MSFsc, and mid-sleep and time in bed on school days (in that order) as independent variables. Model 1 considered only MSQ scores as a predictor (R^2^ = 24.1%; p < 0.001); model 2, MSQ scores and the MSFsc as predictors (R^2^ = 30.7%; p < 0.001); model 3, MSQ scores, the MSFsc, and mid-sleep on school days as predictors (R^2^ = 31.5% ; p = 0.026); and model 4, MSQ scores, the MSFsc, and mid-sleep and time in bed on school days as predictors (R^2^ = 32.2% ; p = 0.04). Model 4 proved to be the superior model (R^2^ = 0.008; p = 0.04), resulting in a statistically significant model [F (4.348) = 42.724; p < 0.001; R^2^ = 0.329]. Thus, we can claim that MSQ scores (β = 0.417; t = 9.194; p < 0.001), the MSFsc (β = 0.174; t = 2.851; p = 0.005), mid-sleep on school days (β = 0.138; t = 2.257; p = 0.025) and time in bed on school days (β = -0.091; t = -2.067; p = 0.04) were predictors of PDSS scores. By comparing the variables included in model 4, we could observe that the time in bed on school days had a reduced predictive role for daytime sleepiness (β = -0.091).

**Table 3:** Linear regression between PDSS score, MSQ score, MSFsc, mid-sleep on school days, and time in bed.

	PDSS (%)^a^	β	Min.	Max.	p
Model 1					
MSQ	24.1	0.471	0.292	0.424	< 0.00^a^
Model 2					
MSQ	30.7	0.435	0.251	0.381	< 0.001^a^
MSFsc		0.268	0.621	1.243	< 0.001^a^
Model 3					
MSQ	31.5	0.423	0.243	0.373	< 0.001^a^
MSFsc		0.175	0.191	1.030	0.004^a^
Mid-sleep (school days)		0.137	0.074	1.156	0.026^a^
Model 4					
MSQ		0.417	0.238	0.368	< 0.001^a^
MSFsc	32.2	0.174	0.188	1.023	0.005^a^
Mid-sleep (school days)		0.138	0.080	1.157	0.025^a^
Time in bed (school days)		-0.091	-0.739	-0.018	0.040^a^

PDSS: pediatric daytime sleepiness scale; MSQ: mini-sleep questionnaire.

Note: MSFsc: considered as an estimate of the subjects’ chronotypes; Mid-sleep on school days: the average point between the time of going to bed and waking up on school days.

^a^ Adjusted R Squared.

^b^ p < 0.05

## DISCUSSION

In this study, we could observe the occurrence of daytime sleepiness in subjects whose average time in bed was in accordance with the recommendations of the National Sleep Foundation (headquartered in Virginia, USA) for their age group^26^. By a hierarchical multiple regression analysis, we could observe that the perception of sleep quality, mid-sleep on school days, and the MSFsc could be variables with greater predictive power for daytime sleepiness in our sample than time in bed. Unlike what we might expect, based on previous studies describing associations between short sleep duration and daytime sleepiness^27,28^, a longer time in bed was insufficient to prevent EDS. According to what Meyer et al.^23^ (2018) proposed, more than half (52.1%) of the students in our sample had EDS (PDSS scores equal to or greater than 15 points). Also, in line with this same study, low sleep quality was the parameter most strongly associated with the occurrence of daytime sleepiness^23^.

This study confirms that daytime sleepiness may correlate with factors other than inadequate sleep duration^28,29^. Thus, our findings are congruent with those in Ferrari Junior et al.^30^ (2019) since these authors found no correlations between daytime sleepiness, as the PDSS assesses, and length of time in bed. The authors found that the perceived need for more sleep and mid-sleep on school days were predictive of daytime sleepiness^30^. In our data, the perception of sleep quality showed the best predictive power for daytime sleepiness, reinforcing the idea that sufficient or ideal sleep covers different aspects - many of which are subjective and susceptible to change throughout life - in addition to time in bed. Owens et al.^31^also emphasized the idea that factors other than sleep duration influence daytime sleepiness. In a literature review, Felden et al.^32^(2015), drew attention to the occurrence of associations between socioeconomic indicators and sleep quality in adolescents. Malheiros et al.^19^ (2021), in a recent survey of Brazilian students (16.4 ± 1.2 years) found an association among daytime sleepiness and low level of physical activity, high consumption of processed foods, and high use of social media.

Regarding sleep quality, we observed that the percentage of students in the sample who obtained scores consistent with poor sleep quality was higher than that observed in other studies. In their validation study of the MSQ scale, Falavigna et al.^25^ applied the scale to a group of 1,108 students with an average age of 22 years and found that 40.4% of their subjects had a score consistent with good sleep quality, with an average score of 26 points. A study by Cayres et al.^33^ (2019) showed that among 120 students aged between 11 and 14 years who answered the MSQ scale, 52.1% had a score equal to or greater than 25 points, which the authors considered as poor sleep. Compared with these two studies, our sample had a lower percentage of subjects with good sleep quality (29%) and a higher mean MSQ score (29.3 points). Although the authors’ data on sleep quality diverged from ours, note that 60.5% of the Brazilian students in the study by Meyer et al.^23^ (2018) perceived their quality of sleep as poor.

In addition to the perception of sleep quality, the MSFsc and mid-sleep on school days also showed predictive power for daytime sleepiness. In our study, we found positive correlations between PDSS scores and mid-sleep point, as well as between PDSS scores and the MSFsc. Thus, we can infer that subjects with greater daytime sleepiness showed a tendency for eveningness. Previous studies observed similar results^31,34,35^. Liu et al.^35^ (2019) conducted a study with 10,086 Hong Kong students and observed that an evening chronotype was the factor most strongly associated with EDS. Martin et al.^34^ (2016) compared morning-type with evening-type students who attended school in different schedules. Both in morning and afternoon schedules, evening-type students had higher PDSS scores than morning-type ones. Owens et al.^31^ (2016) also found a correlation between daytime sleepiness and morningness/eveningness scale for children scores regarding afternoon preferences.

In addition to the chronotype, the occurrence of sleep inertia could be associated with daytime sleepiness observed in our subjects. Roenneberg et al.^36^ (2003) verified the occurrence of sleep inertia in their subjects. In this study, sleep inertia was higher in evening chronotypes, but it was also associated with shorter sleep duration. Although our study ignored the evaluation of sleep inertia, we cannot dismiss the possibility that evening-type subjects start classes under the effect of sleep inertia - which reflect higher PDSS scores. According to previous studies, sleep inertia could take up to two hours to dissipate^37^.

Thus, our results draw attention to the fact that daytime sleepiness is associated with variables other than sleep duration. Therefore, interventions aimed at reducing it, especially in school environments, need to pay attention to this wide range of factors associated with daytime sleepiness. Our data reflect the sleep patterns and daytime sleepiness of a sample of public school students, many of them located on the outskirts of the municipality in which this study was conducted. Many students reported belonging to an economic class whose family income was below 4 minimum wages per month. The findings of Felden et al.^32^ (2015) reinforce that our results represent the sleep patterns and daytime sleepiness of students from lower economic classes, which is an important point for the study of possible intervention measures in Brazil. Due to these results, we suggest that further studies should follow in this direction.

Bearing this socioeconomic scenario in mind, we must mention the limitations of our study, such as our lack of data on living conditions, sleeping places, food, and patterns of physical activity. Moreover, future studies should consider obtaining data on informal^38^ and domestic work, living conditions, and family members’ work shift regimens since they could impact children and adolescents’ quality of sleep and temporal allocation of nighttime sleep episodes.

Moreover, our results serve as an important warning: delaying school start times is a necessary but insufficient intervention to reduce daytime sleepiness and consequently improve academic performance in adolescents. Evening-type adolescents seem to suffer more from daytime sleepiness even when they have the opportunity to extend the duration of their sleep. Maybe some of them could benefit from sleep education and improve their sleep times. Others may need to attend classes even later, starting in the middle of afternoon. The improvement of sleep education programs - seeking long-term effects - and the formulation of a plan to implement schools with flexible times in a digital world have emerged as alternatives for moving toward more inclusive schools.
